# How Authoritarian Leadership Affects Employee's Helping Behavior? The Mediating Role of Rumination and Moderating Role of Psychological Ownership

**DOI:** 10.3389/fpsyg.2021.667348

**Published:** 2021-09-06

**Authors:** Muhammad Asim, Liu Zhiying, Muhammad Athar Nadeem, Usman Ghani, Mahwish Arshad, Xu Yi

**Affiliations:** ^1^School of Management, University of Science and Technology of China, Anhui, China; ^2^International Institute of Finance/School of Management, University of Science and Technology of China, Anhui, China; ^3^Department of Business Administration, Iqra University, Karachi, Pakistan; ^4^College of Education, Zhejiang University, Zhejiang, China; ^5^Department of Economics, Government College Women University, Faisalabad, Pakistan

**Keywords:** authoritarian leadership, helping behaviors, rumination, psychological ownership, COR theory

## Abstract

Interpersonal helping behaviors, i.e., voluntarily assisting colleagues for their workplace related problems, have received immense amount of scholarly attention due to their significant impacts on organizational effectiveness. Among several other factors, authoritarian leadership style could influence helping behavior within organizations. Furthermore, this relationship could be mediated by workplace stressor such as rumination, known as a critical psychological health component leading to depressive symptoms, hopelessness and pessimism. In the meantime, less research attention has devoted to probe the crucial role of psychological ownership, which can buffer the adverse effects of authoritarian leadership upon rumination. Building on conservation of resources theory, this study investigates the adverse impacts of authoritarian leadership on employees' helping behaviors through mediating role of rumination, and also examines the moderating effect of psychological ownership between the relationship of authoritarian leadership and rumination. The data were collected from 264 employees in education and banking sectors and the results show: (i) authoritarian leadership has adverse impacts on helping behavior, (ii) rumination mediates the relationship between authoritarian leadership and employees' helping behaviors, and (iii) psychological ownership moderates the positive relationship between authoritarian leadership and rumination. This study concludes that authoritarian leadership has adverse impacts upon helping behavior, which needs to be controlled/minimized. The findings are of great significance for managers, employees, and organizations in terms of policy implications. The limitations and future research directions are also discussed.

## Introduction

Katz (1964) states that discretionary behaviors are of great importance for organizational effectiveness. Smith et al. (1983) labeled such behaviors as “organizational citizenship behaviors” (OCB). Similarly, helping behavior, defined as “voluntarily helping others with, or preventing the occurrence of, work related problems” (Podsakoff et al., 2000, p. 516), is considered as a facet of organizational citizenship behaviors (OCB) and it is affiliative, cooperative, and directed toward other individuals (Van Dyne and LePine, 1998; Flynn, 2006) and enhances the overall organizational performance (Organ et al., 2005; Liu et al., 2020).

The extant literature have identified several determinants of OCB such as perception of organizational politics (Khan et al., 2019), emotional intelligence (Lim et al., 2018), perceived organizational support (Dai et al., 2018), and psychological contract fulfillment (Ahmad and Zafar, 2018; Mostafa, 2018). However, less in known about whether negative leadership styles (e.g., authoritarian leadership) can effect helping behaviors as these behaviors are more discretionary as compared to other citizenship behaviors (Jex et al., 2003). The potential mechanism for this relationship is not explored. This study aims to address this research gap by investigating the relationship between authoritarian leadership and helping behavior among employees.

Practitioners report that leaders play a critical role in shaping employees' behaviors (Yukl, 2010). The effect of support from supervisors on employees' discretionary behaviors has already been studied in the literature (Eisenberger et al., 2001). For instance, transformational leadership improves discretionary behaviors and is positively associated with organizational commitment, and authentic leadership enhances positive psychological capacities of employees (Walumbwa et al., 2008; Liu and DeFrank, 2013). Moreover, scholars deem management support and empowering leadership as an important expediter toward discretionary behaviors (Srivastava et al., 2006).

Contrastingly, abusive supervision negatively affects such discretionary behaviors as organizational citizenship behaviors (Lyu et al., 2016). Employees facing hostility from their supervisors display increased levels of stress, lower job satisfaction and commitment (Tepper, 2000; Duffy et al., 2002). Authoritarian leadership is a destructive or dark leadership style (Aryee et al., 2007), which may bring several adverse consequences not only for employees but also for organizations. In addition, some studies have revealed that authoritarian leadership is positively related to job attitudes and firms' revenue (Huang et al., 2015) and it is not always responsible for radically reverse conditions (Karakitapoglu-Aygün et al., 2021). Despite this mixed and inconclusive findings of authoritarian leadership, very little is known about the relationship between authoritarian leadership and employees' helping behaviors, and this study aims to fill in the research gap.

Further, the presence of authoritarian leadership may make it difficult for employees to psychologically detach themselves from thinking about the problems at work, which may lead to rumination. Helping others at work requires additional energy and time (Cabrera and Cabrera, 2005), however rumination may be a possible threat for valuable resources, which may affect helping behaviors among employees. This study introduces rumination as a mediating mechanism connecting authoritarian leadership and employees' helping behaviors.

Moreover, previous studies documented that psychological ownership is a key predictor of people's attitudes and behaviors (Hameed et al., 2019). Pierce et al. (2003) state that psychological ownership encourages responsibility containing protective and compassionate feelings for the organization. Psychological ownership, positively associated with citizenship behaviors, job satisfaction and commitment, and negatively related to deviant work behaviors (Avey et al., 2009), could be used as a boundary condition for the relationship between authoritarian leadership and rumination, and can buffer the adverse effects of authoritarian leadership on rumination and weakens their positive relationship.

Competitive advantages in the business environment primarily depend on employees' attitudes and behaviors toward their work and organizations. Along with several other factors, an effective workplace environment through positive leadership is critical to motivate and influence employees to fulfill organizational objectives (Akcin et al., 2017). Positive leadership styles are helpful to boost several types of discretionary behaviors from employees (Srivastava et al., 2006; Liu and DeFrank, 2013). Nevertheless, awful leadership styles could affect such discretionary behaviors as helping behaviors, a type of individual-oriented organizational behaviors with more discretion as compared to other extra-role behaviors (Jex et al., 2003).

## Research Context and Contributions to Literature

Pakistan is a high power distance society as compared to United States and European Union countries (Peltokorpi, 2019). Some studies reported that authoritarian leadership discourage extra-role behaviors in the Pakistani context (Ahmad Bodla et al., 2019; Nazir et al., 2020). Moreover, authoritarian leadership styles have characterized some power distance societies and collectivistic cultures (Chan et al., 2013; Ahmad Bodla et al., 2019). In addition, practitioners also revealed that employees in high power distance contexts are more likely to pattern their behaviors after their superiors (Lian et al., 2012), thus such leadership styles negatively influence employees' behaviors (Chen et al., 2014). Authoritarian leadership practice is among the main reasons behind employees' less involvement in extra-role behaviors in the shape of helping behaviors (Ahmad Bodla et al., 2019). Similarly, it is almost impossible for employees with lower mobility to quit their organizations as they are very much dependent on their current organizations and are not likely to involve in discretionary behaviors (Carpenter and Berry, 2017), as this is the only coping strategy for them to protect themselves from further resource loss.

**Figure 1:**
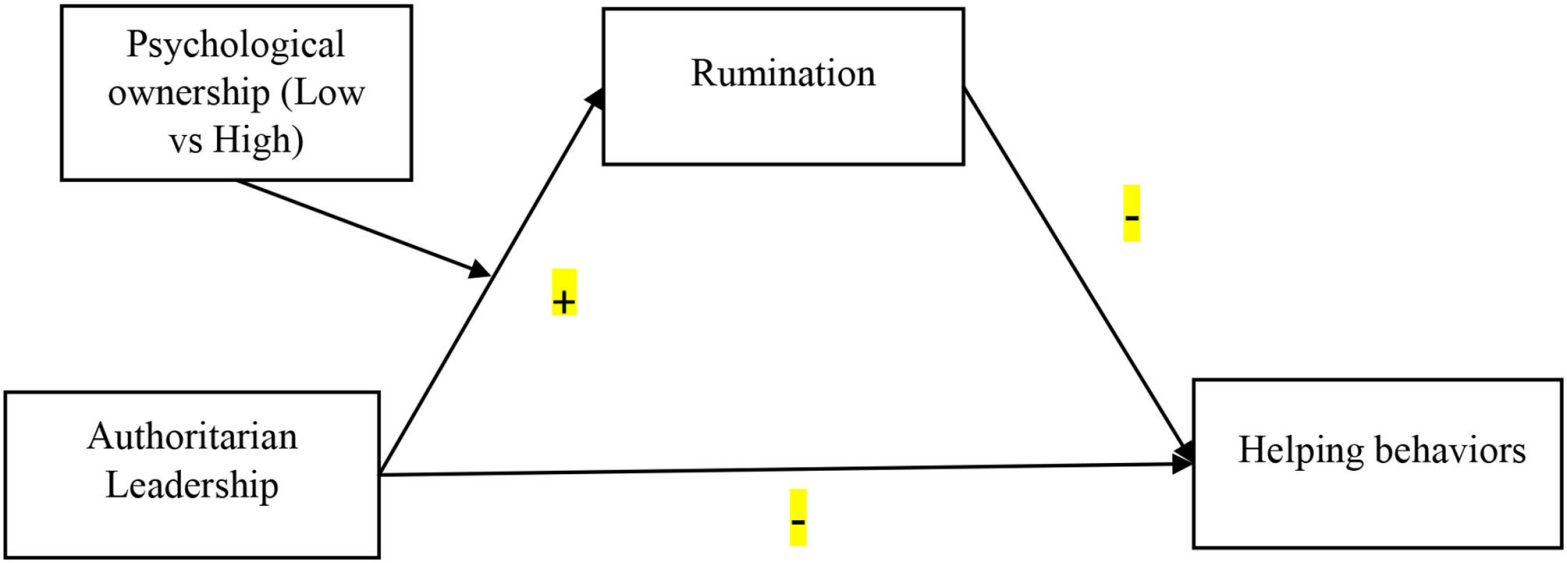
Conceptual framework.

This study contributes to literature in two aspects. First, a moderated mediation model of authoritarian leadership and employees' helping behaviors is constructed, where rumination serves as a mediator and psychological ownership as a moderator. Second, education and banking sectors from a developing country like Pakistan, will further add to the novelty of this paper. Earlier, Wu et al. (2020) have conducted their study in a power distance society (i.e., Taiwan), which shows that authoritarian leadership brings fear among employees, diminishes psychological safety, and discourages extra-role behaviors. However, our study is distinct from their study in the following ways: (i) this study focuses on respondents from various private banks and private education institutions of Pakistan; (ii) the proposed model contains different mediators and moderators (see Figure 1).

## Theoretical Background and Hypothesis Development

### Conservation of Resources Theory

Conservation of resource (COR) theory (Hobfoll, 1989) is a theoretical lens to investigate how authoritarian leadership influences helping behavior among employees. Resources are described as important entities with personal characteristics, objects, energy and conditions (Hobfoll, 1989), and physical and psychological well-beings of employees heavily depend on these valuable resources (Hobfoll, 1989). The COR believes that social relationships are crucial for individuals, and can enhance or deplete the resources and that resource loss is comparatively more prominent than resource gain (Hobfoll and Freedy, 1993), implying that resource loss is more taxing for employees than resource regain. Similarly, scholars report that resource conservation is more important than resource gain, particularly when the subject is going through stress in the shape of resource loss (Halbesleben, 2010). Whenever there is a threat to these resources or the resources are lost, individuals will demonstrate such stress reactions (strains) (Halbesleben et al., 2014) as rumination. Employees who experience authoritarian leadership behavior may prone to fear, strain and feel oppressed (Chan et al., 2013). Leadership support is a valuable job resource which can enhance discretionary behaviors (Kim et al., 2010). However, on the other hand, authoritarian leaders exert absolute power and control over their employees and normally been viewed as a dysfunctional leadership style, therefore employees experiencing authoritarian leadership behaviors are hardly receive any support from their leaders (Lee et al., 2018). Thus, as a dysfunctional leadership style, authoritarian leadership might be viewed as a kind of resource loss (Lee et al., 2018).

Helping is an essential resource of employees. While experiencing resource loss, they become risk-averse in resource investment (Hobfoll, 2001), strategic for resources utilization (Halbesleben, 2010) and eager to protect resources (Halbesleben and Bowler, 2007). Our study assumed that the victims of authoritarian leadership may be reluctant to devote extra time, energy, effort, and other resources toward discretionary actions like helping behaviors in order to avoid further resource loss. COR theory has been widely applied in various research perspectives (Zhang et al., 2017; Lee et al., 2018; Liu W. et al., 2019). For example, Lee et al. (2018) examined the adverse impacts of dark leadership on knowledge sharing behavior by taking emotional exhaustion as mediator and their results revealed that abusive supervision has a positive relationship with emotional exhaustion and reduces the knowledge sharing behaviors among employees. Liu W. et al. (2019) took insight from the COR theory and explored the connection between workplace incivility and organizational citizenship behavior by applying burnout as a mediator. They concluded that workplace incivility significantly leads to burnout and lessens the organizational citizenship behaviors.

The COR theory is particularly useful in our understanding of poor helping behaviors under authoritarian leadership, as these behaviors, compared to in-role behaviors systematically observed and compensated, are more influenced by the levels of personal resources (Halbesleben and Bowler, 2007). Additionally, this study probes into the mechanism under which authoritarian leadership influences employees' helping behaviors. The victims of authoritarian leadership suffering from resource depletion and emotional exhaustion may reduce their energy to focus on their work and resort to rumination and disengagement from voluntary helping behaviors. Helping behaviors require additional efforts and resources (energy). Employees going through rumination are likely to minimize their resource loss and gain control over situations by limiting their participation in helping behaviors.

### Authoritarian Leadership and Employees' Helping Behavior

Helping behaviors are not part of the official contract, but fruitful for organizations, because the employees within organizations voluntarily assist each other (Van Dyne and LePine, 1998). The extant literature showed that positive leadership styles (i.e., transformational leadership and empowering leadership) enhance citizenship behaviors among employees (Srivastava et al., 2006; Liu and DeFrank, 2013). Conversely, such negative leadership styles as authoritarian leadership are negatively related to various important attitudes and behaviors of employees: trust, voice, performance and citizenship behaviors (Pellegrini and Scandura, 2008; Chan et al., 2013; Chen et al., 2014; Shaw et al., 2020).

Authoritarian leaders exercise absolute authority and control along with unquestionable obedience (Cheng et al., 2004; Harms et al., 2018). Authoritarian leadership draw the boundaries of discretion and employees are not allowed to cross those boundaries while performing their work (Aryee et al., 2007). Employees bear the consequences if they fail to follow the rules and procedures set by the authoritarian leadership (Chen et al., 2014). Authoritarian leadership view their employees incapable of performing work without their directions, which deteriorate the self-image of their subordinates (Wu et al., 2002) and these employees may consider authoritarian leadership as a undesirable leadership style and a threat to their self-identities.

Authoritarian leadership style is condemned by researchers because such a style possesses extremely directive or commanding behaviors attempting to control subordinates (Shen et al., 2019). The extant literature states that authoritarian leaders often control their employees through threats and intimidation (Kiazad et al., 2010), forming strong connections with employees' negative emotions such as anger and fear (Farh et al., 2014; Guo et al., 2018). Authoritarian leadership style containing some features different from abusive supervision is described as a display of non-physical hostility toward their subordinates (Tepper, 2000). Not all authoritarian leaders are abusive. Our study aims to examine the relationship of authoritarian leadership with positive behaviors (e.g., helping behaviors), which is still unexplored in the extant literature. Recent research suggests that employees will be less involved in extra-role behaviors when they experience authoritarian leadership (Ahmad Bodla et al., 2019).

It is assumed that authoritarian leadership impedes employees' motivation toward positive behaviors and results in lower helping behaviors among employees. Thus, the following hypothesis is proposed:

**Hypothesis 1**. Authoritarian Leadership adversely affects helping behaviors among employees.

### Mediating Effect of Rumination

The mediating role of rumination is also a building block of this study. Rumination is defined as negative and persistent thoughts and actions on symptoms and outcomes of past personal experiences (Nolen-Hoeksema, 1991, 2000). The cognitive pattern depends on previous events and outcomes of frustrations confronted by employees (Aldao et al., 2010). Scholars report that rumination is the result of traumatic events containing three key steps (Watkins, 2008). To begin with, rumination produces adverse feelings and emotions. Furthermore, problem solving intentions and mood regulations are obstructed by those feelings and emotions. Finally, rumination boosts generalization beyond the event due to its self-referential in nature.

Rumination is associated with poorer concentration, lower levels of motivation and cognition, reduced problem solving abilities, higher levels of stress, and more difficulties in social relationships (Lyubomirsky and Tkach, 2008; Bortolon et al., 2019; Karabati et al., 2019). It is documented that rumination is positively related with emotional exhaustion (Donahue et al., 2012), and poor performance (Shapiro, 2013). Self-referential nature of ruminators deprived them from thinking out of the box. It's hard to divert their attention from self-related information and negative appraisals of the self-emotions to non-self-related information, so the ruminators display poor psychological state (McKie et al., 2017). Rumination makes employees unhappy by creating discrepancies between their current and desired states (Watkins, 2008) and these employees are considered unhelpful to their peers or their organizations (Watson and Clark, 1984).

Scholars found that inspirational and visionary leadership in the shape of transformational leadership expresses a higher work purpose to the subordinates, fulfills their intrinsic needs and motivates them (Judge and Piccolo, 2004), and results in a lower level of rumination about work (Perko et al., 2016). In contrast, unfair treatment from leadership is likely to instigate ruminative thoughts due to experience of stress-production (Ford and Huang, 2014; Tarraf et al., 2019). According to the COR theory, increase in work demands may force employees to use resources to cope with such demands, which may cause strain reaction in the form of rumination. Employees will experience rumination when essential resources are under threat as a result of authoritarian leadership. Extra-role behaviors are more influenced by personal resources (Halbesleben and Bowler, 2007). Hence rumination may compel the employees under authoritarian leadership to engage in less helping behaviors, just as a countermeasure against continuous depletion of resources. Thus, Hypothesis 2 is proposed.

**Hypothesis 2**. Rumination mediates the relationship of authoritarian supervision and helping behaviors.

### Moderating Effect of Psychological Ownership

Ownership is a prime motivator of human behavior (Liu F. et al., 2019). Psychological ownership is “the state in which individuals feel as though the target of ownership or a piece of that target is theirs” (Pierce et al., 2003, p. 86). Scholars consider psychological ownership as an important construct in shaping the employees' responses toward organizations (Pierce et al., 2009; Chen et al., 2021). It is associated with pride and responsibility, and encourages employees to exhibit behaviors which are not part of their official contract (Van Dyne and Pierce, 2004). Feeling of ownership can be developed toward tangible and intangible targets in the organizational context, for example, a novel idea, specific project or the organization (Liu F. et al., 2019). Avey et al. (2009) proposed organization to be the most important target of Psychological ownership and revealed that psychological ownership is a feeling with which employees make decision that are in the best interest of the organization. Feeling of ownership toward their respective organizations is the main theme of this study. With this kind of ownership or possession, employees become psychologically tied to their organization (Pierce and Jussila, 2011) and make decisions which are in the best interest of the organization (Avey et al., 2009). Emotionally depleted employees with less involvement with others at work display negative attitudes and behaviors which can be reduced with the help of psychological ownership (Kaur et al., 2013). Moreover, higher psychological ownership helps employees to mitigate the adverse impacts of dark leadership. Hence, their reactions are thoughtful and inclined toward the target (organization) while they face workplace stressors (Ghani et al., 2020). According to COR, employees facing aggression at work are less involved in the activities harming the organization if they have higher psychological ownership, as the resource loss caused by aggression could be reduced by the buffering provided through resource of psychological ownership (Kong and Kim, 2017).

Based on the above literature, it is reasonable to suppose that the relationship of authoritarian leadership with rumination may be weakened through such individual characteristics as psychological ownership. Therefore, this study assumes that the positive relationship between authoritarian leadership and rumination will be weaker for employees with higher levels of psychological ownership. The employees with higher psychological ownership are motivated toward goals and are expected to be more thoughtful and cautious in their response toward stressors. Hence, the following hypothesis is proposed.

**Hypothesis 3(a):** The authoritarian leadership is strongly positively associated with rumination when psychological ownership is low (vs. high).

A moderation mediation hypothesis is also developed for this study. The expectation that authoritarian leadership can be positively associated with rumination when ownership is low (vs. high) and, in turn, rumination could be associated with employees' lower helping behaviors. Hence, we propose the following:

**Hypothesis 3(b)** The negative indirect relationship between authoritarian leadership and employees' helping behaviors through rumination will be stronger for low (vs. high) levels of psychological ownership.

## Materials and Methods

### Participants and Procedures

Data were collected from 264 employees working in Pakistani banking and education organizations. Diversified samples from various organizations enhance the generalizability of our results (Highhouse, 2009). Initially, we approached human resource managers from each organization, described our academic purpose, and requested them to help us by providing a list of employees having at least 1-year of working experience with their respective bosses. Additionally, surveys were distributed in English to employees through the support of managers. The cover letters pointed out the basic research purpose and assurance of confidentiality of their responses. Figure 1 shows the proposed research framework of the study.

To minimize the chances of common method variance issue (Podsakoff et al., 2003), the data were collected at different times. A time lag of 3 weeks was considered while the questionnaires were distributed. To match time 1 (T1), time 2 (T2), and time 3 (T3) surveys, a distinctive identifier was assigned to each participant response. The anonymity of the respondents was ensured via a unique identifier comprising of only a numerical number to match the responses of T1, T2 and T3 rather than personal information of the respondents. At T1, data of 372 respondents were collected for the first set of 408 questionnaires containing demographics and independent variable (authoritarian leadership) items. For the second round (T2), questionnaires sent to those who had responded in T1 included moderating (psychological ownership) and mediating (rumination) variables, and data of 338 participants were obtained. Finally, at T3, questionnaires for those who had responded in T2 employed the dependent variable (employees' helping behaviors). 296 responses were received at T3 and the response rate was 72.55%. In the final set of responses, 32 questionnaires were uncompleted or not properly filled, and thus excluded. 264 valid samples were used to carry out the study analysis. The respondents' demographics are given in Table 1.

**Table 1:** Respondents information.

**Variables**	**Categories**	**Number**	**Percentage**
Gender	Male	131	49.6
	Female	133	50.4
Age (in years)	<25	100	37.9
	25–30	113	42.8
	31–35	35	13.3
	>35	16	6.1
Education	High school	60	22.7
	Bachelor	126	47.7
	Masters	68	25.8
	Post master	10	3.8
Work experience (in years)	<1	20	7.6
	1–5	127	48.1
	6–10	64	24.2
	>10	53	20.1

### Measures

Participants were asked to respond to each item via a five-point Likert scale. The scale ranged from 5 (= strongly agree) to 1 (= strongly disagree).

#### Authoritarian Leadership

The construct of authoritarian leadership was measured via 9 items adapted from Farh and Cheng (2000). One sample item of the scale is “My supervisor asks me to obey his/her instructions completely” (Cronbach's alpha = 0.87). The model fitness indices were good as X^2^/df= 2.810, *p* < 0.000, CFI = 0.983, and RMSEA = 0.080. The factor loadings of the items ranged from 0.735 to 0.875.

#### Psychological Ownership

Psychological ownership was measured *via* 7 items from Van Dyne and Pierce (2004). One sample item of this scale is “I sense that this organization is OUR company” (Cronbach's alpha = 0.93). The model fitness indices were good as X^2^/df= 2.426, *p* < 0.000, CFI = 0.994, and RMSEA = 0.074. The factor loadings of the items ranged from 0.766 to 0.845.

#### Rumination

Rumination was measured via the 12-item scale adapted from Trapnell and Campbell (1999). The sample item of this scale is “I tend to ruminate or dwell over things that have happened to me for a really long time afterward” (Cronbach's alpha = 0.90). The model fitness indices were good as X^2^/df= 2.878, *p* < 0.000, CFI = 0.965, and RMSEA = 0.084. The factor loadings of the items ranged from 0.703 to 0.894.

#### Helping Behavior

Helping behavior was measured via 7 items adapted from Van Dyne and LePine (1998). One sample item is “I used to help others in their work responsibilities” (Cronbach's alpha = 0.95). The model fitness indices were good as X^2^/df= 2.890, *p* < 0.000, CFI = 0.985, and RMSEA = 0.085. The factor loadings of the items ranged from 0.705 to 0.852.

### Control Variables

Previous studies presented some influence of demographic variables on employees' helping behaviors, i.e., gender, age, education and work experience (Randel et al., 2016; De Clercq et al., 2019). Therefore, this study controls these factors which may influence the chosen variables of this study.

### Analytical Approach

Several statistical tools were employed for data analysis via SPSS and AMOS. The composite reliability (CR), factor loading, Cronbach's alpha, and average variance extracted (AVE) were assessed to evaluate the reliability and validity of the research model. Further, model fitness indices like X^2^ (CMIN/df), RMSEA, and comparative fit index (CFI) were examined (Hu and Bentler, 1999; Hair et al., 2010; Islam et al., 2020). The Process Macro (Hayes, 2013) is adopted to test the moderation and mediation effects (Ghani et al., 2020). The process Macro has also been used in the recent studies to the test the similar models (Kiani et al., 2020, 2021; Usman et al., 2020; Halima et al., 2021).

## Results

### Measurement Tests

Although the data for this study were collected in time lags, the threat of common method bias (CMB) was assessed in light of Podsakoff et al. (2003). Two approaches were employed to study CMB. First, the Harman single-factor test was adopted and the findings revealed that one factor explained the total variance 36.18%, which is within the cutoff value of 50% (Harman, 1967). Therefore, CMB is not a subject of concern in this research. Second, CMB occurs when the inter-correlations among the study variables are higher than 0.90. **Table 3** showed that the inter-correlation between the variables are within the suggested range of 0.90. Thus, the two approaches demonstrated that CMB was not a potential issue of this study.

### Hypothesis Test

Table 2 demonstrates the correlations among the variables, and are in the expected directions. The PROCESS macro (Hayes, 2013) was employed to examine both direct and indirect effects to test H1, H2, H3a, and H4b. Table 3 reveals that authoritarian leadership has an adverse significant effect on employees' helping behaviors (β = −0.306, *t* = −7.461, *p* < 0.001), confirmed H1. Further, rumination mediates the relationship between authoritarian leadership and employees' helping behaviors as CI (−0.1239, −0.0245) did not include zero, and H2 is also confirmed.

**Table 2:** Descriptive results.

	**Mean**	**SD**	**1**	**2**	**3**	**4**	**5**	**6**	**7**	**8**
1. Gender	1.50	0.50	1							
2. Age	1.88	0.86	−0.206^**^	1						
3. Education	2.11	0.79	−0.173^**^	0.293^**^	1					
4. Work experience	2.57	0.89	−0.111	0.103	0.123^*^	1				
5. AL	2.66	1.08	0.062	0.043	−0.058	0.016	**(0.829)**			
6. PO	3.26	0.87	−0.036	−0.029	−0.107	−0.082	−0.308^**^	**(0.809)**		
7. RU	2.33	0.96	−0.039	0.018	−0.107	−0.065	0.346^**^	−0.381^**^	**(0.795)**	
8. HB	3.43	0.79	0.062	−0.069	−0.013	−0.072	−0.419^**^	0.249^**^	−0.360^**^	**(0.773)**

*n = 264*,

** *p < 0.01*.

* *p < 0.05*.

*AL, authoritarian leadership; PO, psychological ownership, RU, rumination; HB, helping behavior. The bold values presented in parentheses indicate discriminant validities*.

**Table 3:** Mediation effect.

	**β (unstandardized)**	**SE**	**t**	***p***	***LL95%CI***	***UL95%CI***
**Outcome: employees' helping behavior**						
Constant	4.247	0.118	36.024	0.000	4.0151	4.4794
Authoritarian leadership	−0.306	0.041	−7.461	0.000	−0.3873	−0.2255
**Outcome: Rumination**						
Constant	1.517	0.147	10.296	0.000	1.2265	1.8066
Authoritarian Leadership	0.305	0.051	5.961	0.000	0.2048	0.4068
**Outcome: employees' helping behavior**						
Constant	4.554	0.135	33.626	0.000	4.2876	4.8210
Rumination	−0.203	0.048	−4.225	0.000	−0.2969	−0.1081
Authoritarian leadership	−0.245	0.042	−5.763	0.000	−0.3280	−0.1609
	**Effect**	***SE***	**LL 95% CI**	**UL 95 % CI**		
Indirect effect	−0.061	0.031	−0.1239	−0.0245		
	**Effect**	***SE***	***z***	***p***		
Normal theory test for indirect effect	−0.061	0.018	−3.415	0.001		

*Bootstrap sample size = 5000, CI, confident interval; LU, lower limit; UL, upper limit*.

**Figure 2:**
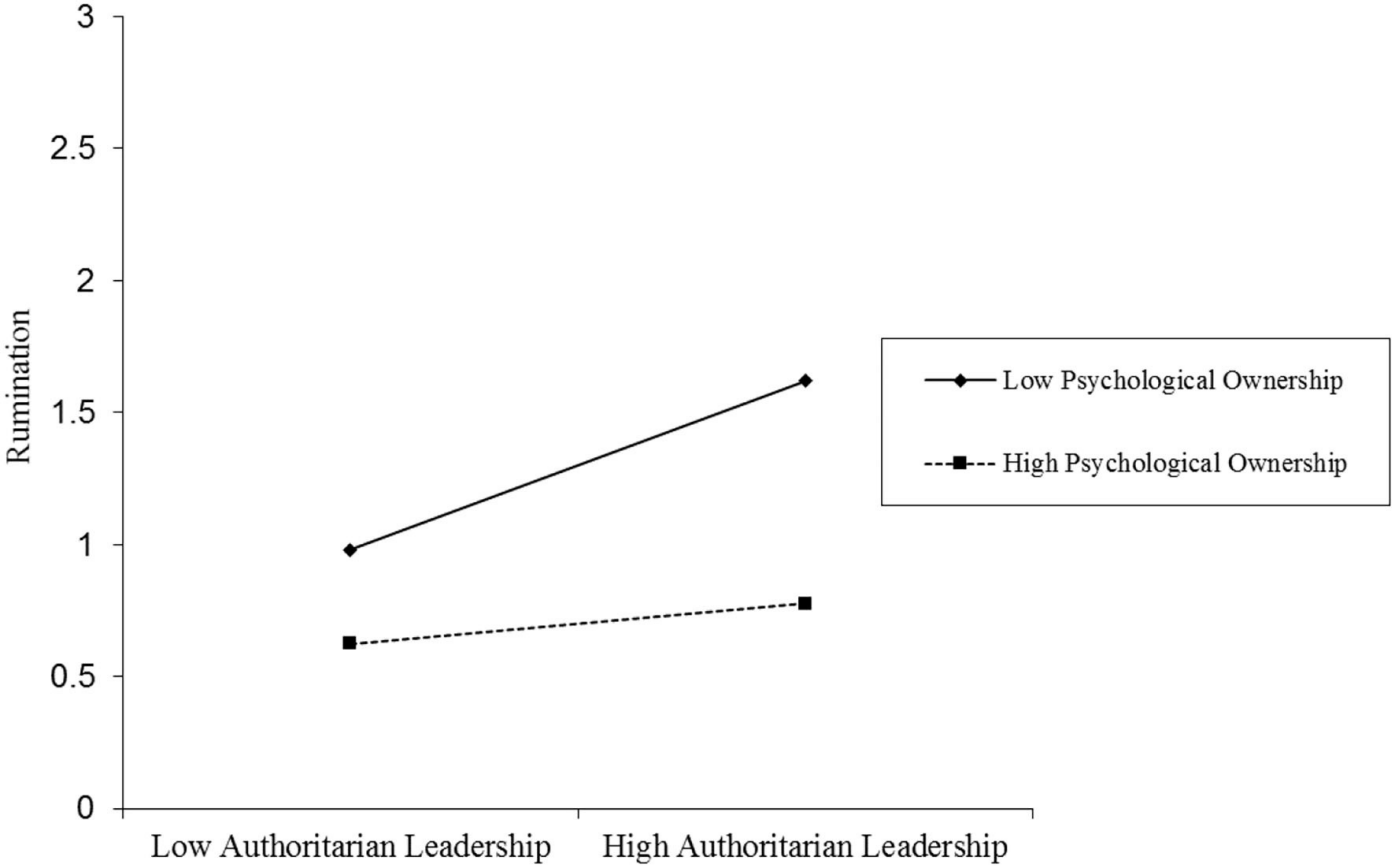
Moderating effect of psychological ownership between the relationship of authoritarian leadership and rumination.

The findings of the moderating role of psychological ownership between the relationship of authoritarian leadership and rumination as reported in Table 4 reveal that the interaction effect (authoritarian leadership x psychological ownership) (β = −0.122, *t* = −2.317, *p* < 0.05) is significant, thus H3a is validated. The moderating effects of psychological ownership are in the graphical form in Figure 2. Further, psychological ownership is split into high (+1 SD) and low (−1 SD) levels to examine the nature of interaction effects. The positive association between authoritarian leadership and rumination is non-statistically significant (β = 0.091, *t* = 1.193, *p* > 0.05, CI = −0.0594 to 0.2423) when psychological ownership is higher. However, this relationship is stronger (β = 0.304, *t* = 4.481, *p* < 0.001, CI = 0.1703–0.4373) when psychological ownership is lower. The results provide support for the moderation hypothesis.

**Table 4:** Moderation effect.

**Outcome: Rumination**	**β (unstandardized)**	**SE**	**t**	***p***	**LL95%CI**	**UL95%CI**
Constant	2.95	0.054	42.436	0.000	2.1881	2.4010
Authoritarian leadership	0.198	0.056	3.531	0.001	0.0874	0.3078
Psychological ownership	−0.301	0.072	−4.198	0.000	−0.4428	−0.1600
Authoritarian leadership × psychological ownership	−0.122	0.052	−2.317	0.021	−0.2264	−0.0183

*Bootstrap sample = 5000, CI, confident interval; LU, lower limit; UL, upper limit*.

For moderated-mediation (see Table 5), the expected indirect effect of authoritarian leadership was assessed on employees' helping behaviors via rumination conditional on levels of psychological ownership (H3b). High and low levels of psychological ownership (±1 SD) were set to evaluate the conditional indirect effect of authoritarian leadership on employees' helping behaviors (via rumination). Table 5 means that the indirect effect is stronger (β = −0.062, CI_95%_ = −0.125 to −0.024) at a lower level of psychological ownership, but weaker (β = -0.019, CI_95%_ = −0.065 to −0.008) at a higher level of psychological ownership, hence, H3a confirmed.

**Table 5:** Moderated mediation model.

	**Indirect effect**	**Boot SE**	**LL95%CI**	**UL95%CI**
Conditional indirect effect at range of psychological ownership = M ± 1SD				
–SD(−0.868)	−0.062	0.025	−0.125	−0.024
M(0.000)	−0.040	0.018	−0.087	−0.014
+SD(+0.868)	−0.019	0.018	−0.065	0.008

*N = 282; 95% confidence intervals for the conditional indirect effects; Bootstrap sample size = 5000; LLCI, lower limit confidence interval; ULCI, upper limit confidence interval*.

## Discussions, Implications, and Limitations

### Discussions

This study explored the untapped relationship of authoritarian leadership and helping behaviors among employees, and rumination as a mediation mechanism was also examined for this relationship. Further, the moderating role of psychological ownership as a boundary condition was employed for the investigation of the relationship between authoritarian leadership and rumination. Besides, the moderated-mediation framework was constructed to investigate the mediating effect of rumination upon the relationship between abusive supervision and helping behaviors on low and high levels of psychological ownership. Based on the COR theory (Hobfoll, 1989), the findings showed that authoritarian leadership has a direct adverse effect on helping behaviors among employees, and this relationship is mediated by rumination. The results also suggest that psychological ownership plays a significant role as a boundary condition between the relationship of authoritarian leadership and rumination and weakens this relationship. Furthermore, it is found that the indirect effect of authoritarian leadership on helping behavior among employees via rumination is contingent on psychological ownership and the adverse effect of authoritarian leadership on employees' helping behavior via rumination attenuated with a higher (vs. low) level of psychological ownership.

### Theoretical Implications

Based on the COR theory (Hobfoll, 1989, 2001), this study makes contributions in the domain of authoritarian leadership and employees' helping behaviors in the following aspects. First, this is a pioneering endeavor probing into the relationship between authoritarian leadership and employees' helping behaviors and the findings reveal that the authoritarian leadership adversely influences the helping behaviors. Previous studies showed that authoritarian leadership has adverse effects on employees' positive attitudes and behaviors, like organizational commitment (Erben and Güneşer, 2008) and organizational citizenship behavior (Chen et al., 2014), employee creativity (Guo et al., 2018), deviant work behaviors (Jiang et al., 2017). Our study adds to the body of literature by demonstrating the detrimental effect of authoritarian leadership on helping behaviors among employees and the results offer an important contribution for organizations.

Second, our study shows that authoritarian leadership is a source of rumination because essential resources are depleted as a result of authoritarian leadership. Individuals experiencing rumination are enjoying less happiness and life satisfaction (Karabati et al., 2019). The extant literature reports that combination of depleted resources and high work demands in shape of authoritarian leadership functions as a breeding ground for ruminative thoughts (Perko et al., 2017).

Third, rumination is a negative psychological state which may exhaust physical vitality. Employees going through high rumination have the tendency to withheld information and ideas at work, as it depletes psychological resources required for contribution in social interactions (Madrid et al., 2015), including helping others at work. Querstret and Cropley (2012) predicted it as a fatigue related to work.

Fourth, this study contributes to the authoritarian leadership literature by investigating the mediating role of rumination that explains the relationship between authoritarian leadership and helping behaviors. Unfair or abusive behaviors on the part of the supervisors can easily turn into real stressors (Perko et al., 2017). In line with the COR, employees suffering from authoritarian leadership experience a stress reaction, and fail to find a solution, resulting in rumination about authoritarian leadership, which further depletes resources, and employees might decrease helping behaviors in order to preserve remaining resources.

Fifth, the role of psychological ownership is examined as a positive individual trait in the relationship between authoritarian leadership and rumination. Employees containing higher psychological ownership are less likely to go through rumination thoughts even under authoritarian leadership. Previous literature showed that a high level of personal trait prevents employees going through mistreatment at work from reacting in ways that compromise their integrity (Roberts et al., 2005). Psychological ownership is also a type of positive personal characteristic helping to eliminate the adverse effects of destructive leadership and decreases strain (rumination).

Finally, a moderated mediation framework is constructed and proves that the mediating effect of rumination fluctuates with the level of psychological ownership, and that the indirect effect of authoritarian leadership on employees' helping behaviors through rumination is stronger when psychological ownership is low (vs. high). Pakistan belongs to a high power distance society as compared to united states and European union countries and authoritarian leadership highly characterizes power distance societies and collectivistic cultures (Chan et al., 2013; Ahmad Bodla et al., 2019). In power distance cultures, authoritarian leadership is very much successful in achieving operational efficiency and performance (Huang et al., 2015). It might be beneficial for core tasks and compulsory duties, but high authoritarian leadership will decreases discretionary behaviors of employees (Ahmad Bodla et al., 2019; Naseer et al., 2020). Thus, our results are consistent with previous findings.

### Practical Implications

Practical implications are put forward for management of firms and organizations.

First, authoritarian leadership styles should be abbreviated by adequately selecting and training the leaders, as authoritarian leadership is related with a number of negative work-related outcomes. Personality characteristic of “dominance” (i.e., the level of assertiveness, cooperation and aggression) measured by Cattell et al. (1970) should be observed as core indicators for the recruiting of leaders or supervisors. Besides, interpersonal skills of the leaders should be improved through training courses (Aryee et al., 2007). More importantly, leaders should endeavor to build a fair and pleasant environment. Existing literature reveals that leaders play a significant role in developing better working outcomes for employees (Klaic et al., 2018), which may bring several positive outcomes encouraging helping behaviors among colleagues.

Second, unfriendly and poor working experiences with leaders influence psychological states and lead to rumination. Strategies should be devised to lower the tendency of rumination thoughts. Unpleasant events like poor social interactions have a greater effect on one's psychological state than positive events (Karabati et al., 2019), as they require more attention, produce more casual attributions difficult to deal with. Managers and employees need to be vigilant of these systematic biases and errors while trying to stop rumination from aggravating. Measures could be taken to reduce the rumination by placing priority on leisure time and acknowledging leisure time as an important time to recover (Richter et al., 2020). Management could organize workshops and seminars to inform employees of the importance of leisure time, and facilitate their diversion of their concentration from ruminative thoughts. Acceptance and commitment therapy could be a useful mechanism in the working environment (Seear and Vella-Brodrick, 2013), which can help individuals learn how to lie in the moment by suppressing thoughts about past or future potential events.

Third, managers need to assist employees in their abilities to cope with workplace stressors and demanding relationships at work. This study proposes that respective management could benefit from helping employees develop strong psychological attributes.

Fourth, the possibility of future positive event appears weaker for employees with high rumination as compared to that of the potential negative event. Hence, employees unsatisfied with their jobs and working under stress may take advantage of keeping a diary of positive events at work in order to divert their attention from negative thoughts or to attenuate effects from them.

Recently, Dawkins et al. (2017) found that it could be possible to improve psychological ownership by making certain structural changes (Personal control over work, accountability, role within a company) in the organization. As prior studies have proved, differences among employees positively affects citizenship behaviors differently (Bogilović et al., 2017; Ghani et al., 2020). Moreover, organizational managers need to improve employees' psychological ownership by creating a supportive organizational culture (Kong and Kim, 2017). For example, it is possible to delegate authority (Pierce et al., 2001) or to redesign the job (Pierce et al., 2009) to increase psychological ownership.

### Limitations and Future Research Directions

This study is subject to some limitations which could pave ways for future research. Firstly, the conceptual framework was investigated in the Pakistani organizational context. Future studies can examine this model in other countries and organizational settings to generalize the findings. More specifically, the future studies may be conducted in Western countries to observe the differences between Asian and Western cultures as mental and behavioral responses vary from culture to culture (Chen, 1995).

Secondly, although a time-lagged design spanning over three periods is adopted, it is hard to claim causality because it is not purely longitudinal. Therefore, future examinations could consider multi-wave longitudinal or experimental designs.

Thirdly, the data of independent variable (authoritarian leadership) and dependent variable (helping behavior) were obtained from a single source (employees), so there might be chances of common method bias (Podsakoff et al., 2003), although a time lagged data collection could help to minimize this bias. As the variables of the study depend on the employees' own perception, we have to trust on the respondents own ability to judge their own feelings while answering the questions (Bader, 2015). Future research could collect data from other sources. For example, authoritarian leaders or coworkers are better sources of data on employees' helping behaviors as they interact with the rated employees daily.

Fourthly, this study investigates psychological ownership as a significant boundary condition mitigating the adverse effect of authoritarian leadership on rumination. Future studies may consider psychological safety, psychological capital, etc.

Finally, future analyses could be conducted of antecedents of authoritarian leadership with other psychological mechanisms (psychological contract breach, psychological capital, psychological security etc.) influencing helping behaviors.

## Conclusion

This study proposes a conceptual framework based on the COR theory and hypothesizes relationship between authoritarian leadership and helping behaviors among employees at workplace. The findings reveal that authoritarian leadership has direct adverse effects on helping behaviors among employees and this relationship is mediated by rumination. The mediating effect is weaker for employees with high psychological ownership as compared to those with lower psychological ownership. This research contributes to literature in the domain of authoritarian leadership and employees' helping behaviors. The conclusions are of strong policy implications for organizations, HR management, and managers/supervisors to ensure healthy working environment through effective management policies and practices.

## Data Availability Statement

The raw data supporting the conclusions of this article will be made available by the authors, without undue reservation.

## Ethics Statement

Ethical review and approval was not required for the study on human participants in accordance with the local legislation and institutional requirements. The patients/participants provided their written informed consent to participate in this study.

## Author Contributions

Conceptualization: MAs and UG. Methodology and formal analysis: UG and MN. Project administration: LZ and XY. Supervision: LZ. Writing—original draft: MAs. Writing—review & editing: MAr, LZ, MN, and UG. All authors have read and agreed to the published version of the manuscript.

## Conflict of Interest

The authors declare that the research was conducted in the absence of any commercial or financial relationships that could be construed as a potential conflict of interest.

## Publisher's Note

All claims expressed in this article are solely those of the authors and do not necessarily represent those of their affiliated organizations, or those of the publisher, the editors and the reviewers. Any product that may be evaluated in this article, or claim that may be made by its manufacturer, is not guaranteed or endorsed by the publisher.
